# COMPLEXITY OF PEDIATRIC CHRONIC DISEASE: CROSS-SECTIONAL STUDY WITH 16,237 PATIENTS FOLLOWED BY MULTIPLE MEDICAL SPECIALTIES

**DOI:** 10.1590/1984-0462/2020/38/2018101

**Published:** 2019-11-25

**Authors:** Caroline Gouveia Buff Passone, Sandra Josefina Grisi, Sylvia Costa Farhat, Thais Della Manna, Antonio Carlos Pastorino, Renata Antunes Alveno, Caroline Vasconcelos Sá Miranda, Aurora Rosaria Waetge, Mariana Nutti Cordon, Vicente Odone-Filho, Uenis Tannuri, Werther Brunow Carvalho, Magda Carneiro-Sampaio, Clovis Artur Silva

**Affiliations:** a Universidade de São Paulo, São Paulo, SP, Brazil.

**Keywords:** Chronic disease, Child, Adolescent, Emergency, Intensive care unit, Hospitalization, Doença crônica, Criança, Adolescente, Emergência, Unidade de cuidados intensivos, Hospitalização

## Abstract

**Objective:**

To assess demographic data and characteristics of children and adolescents with pediatric chronic diseases (PCD), according to the number of specialties/patient.

**Methods:**

We performed a cross-sectional study with 16,237 PCD patients at outpatient clinics in one year. Data were analyzed by an electronic data system, according to the number of physician appointments for PCD. This study assessed: demographic data, follow-up characteristics, types of medical specialty, diagnosis (International Statistical Classification of Diseases and Related Health Problems - ICD-10), number of day hospital clinic visits, and acute complications.

**Results:**

Patients followed by ≥3 specialties simultaneously showed a significantly higher duration of follow-up compared to those followed by ≤2 specialties [2.1 (0.4-16.4) vs. 1.4 (0.1-16.2) years; p<0.001] and a higher number of appointments in all specialties. The most prevalent medical areas in patients followed by ≥3 specialties were: Psychiatry (*Odds Ratio* - OR=8.0; confidence interval of 95% - 95%CI 6-10.7; p<0.001), Palliative/Pain Care (OR=7.4; 95%CI 5.7-9.7; p<0.001), Infectious Disease (OR=7.0; 95%CI 6.4-7.8; p<0.001) and Nutrology (OR=6.9; 95%CI 5.6-8.4; p<0.001). Logistic regressions demonstrated that PCD patients followed by ≥3 specialties were associated with high risk for: number of appointments/patient (OR=9.2; 95%CI 8.0-10.5; p<0.001), day hospital clinic visits (OR=4.8; 95%CI 3.8-5.9; p<0.001), emergency department visits (OR=3.2; 95%CI 2.9-3.5; p<0.001), hospitalizations (OR=3.0; 95%CI 2.7-3.3; p<0.001), intensive care admissions (OR=2.5; 95%CI 2.1-3.0; p<0.001), and deaths (OR=2.8; 95%CI 1.9-4.0; p<0.001). The diagnosis of asthma, obesity, chronic pain, and transplant was significantly higher in patients followed by ≥3 specialties.

**Conclusions:**

The present study showed that PCD patients who required simultaneous care from multiple medical specialties had complex and severe diseases, with specific diagnoses.

## RESUMO

**Objetivo:**

Avaliar dados demográficos e características de crianças e adolescentes com doenças crônicas pediátricas, de acordo com o número de especialidades/paciente.

**Métodos:**

Realizou-se um estudo transversal com 16.237 pacientes com doenças crônicas pediátricas durante um ano. A análise foi feita em um sistema eletrônico, de acordo com número de consultas médicas para doenças crônicas pediátricas. Este estudo avaliou dados demográficos, características do seguimento, tipos de especialidades médicas, diagnóstico (10ª Revisão da Classificação Estatística Internacional de Doenças e Problemas Relacionados com a Saúde - CID-10), número de visitas e complicações agudas.

**Resultados:**

Os pacientes acompanhados por três ou mais especialidades simultaneamente tiveram seguimento de maior duração comparados com aqueles seguidos por ≤2 especialidades [2,1 (0,4-16,4) vs. 1,4 (0,1-16,2) anos; p<0,001], bem como maior número de consultas em todas as especialidades. As áreas médicas mais comuns em pacientes acompanhados por ≥3 especialidades foram: psiquiatria (*Odds Ratio* - OR=8,0; intervalo de confiança de 95% - IC95% 6-10,7; p<0,001); dor/cuidados paliativos (OR=7,4; IC95% 5,7-9,7; p<0,001); doenças infecciosas (OR=7,0; IC95% 6,4-7,8; p<0,001); nutrologia (OR=6,9; IC95% 5,6-8,4; p<0,001). As regressões logísticas mostraram que os pacientes com doenças crônicas pediátricas seguidos por ≥3 especialidades tinham alto risco para: maior número de consultas/paciente (OR=9,2; IC95% 8,0-10,5; p<0,001); atendimentos em hospital-dia (OR=4,8; 95%IC3,8-5,9; p<0,001); atendimentos em pronto-socorro (OR=3,2; IC95% 2,9-3,5; p<0,001); hospitalizações (OR=3,0; IC95%2,7-3,3; p<0,001); internação em terapia intensiva (OR=2,5; IC95% 2,1-3,0; p<0,001); óbitos (OR=2,8; IC95%1,9-4,0; p<0,001). Os diagnósticos de asma, obesidade, dor crônica, transplante e infecção do trato urinário foram mais frequentes nos pacientes seguidos por três ou mais especialidades.

**Conclusões:**

O presente estudo mostrou que pacientes com doenças crônicas pediátricas que necessitaram de múltiplas especialidades médicas simultaneamente apresentavam doenças complexas e graves, com diagnósticos específicos.

## INTRODUCTION

The prevalence of pediatric chronic diseases (PCD) has increased worldwide over the last years. This fact is due to the advancements in medical diagnosis, with new technologies and specific treatments for different illnesses and comorbidities.^1^ ^,^ ^2^ ^,^ ^3^ ^,^ ^4^ ^,^ ^5^ ^,^ ^6^ ^,^ ^7^ ^,^ ^8^ ^,^ ^9^ ^,^ ^10^ ^,^ ^11^

Of note, child and adolescent populations with chronic health disabilities requiring highly complex medical care have been increasingly followed-up in tertiary centers. A French study with individuals younger than 14 years old showed that 3.3% of patients had long-term conditions, and 1.4% had complex chronic diseases.^12^ The outcomes are distinct for these patients and need assessment of multiple specialties.^13^ ^,^ ^14^ ^,^ ^15^ ^,^ ^16^ ^,^ ^17^ ^,^ ^18^ ^,^ ^19^ ^,^ ^20^

Recently, a study evaluated a large population of children and adolescents with PCDs followed in a Latin American tertiary hospital. The authors reported that patients required many appointments in multiple medical specialties and hospital admissions, especially in early adolescence.^1^ However, no systematic analysis based on PCD patients followed simultaneously by multiple specialties and to evaluate etiological diagnosis has been carried out.

Therefore, the objective of the present study was to describe and compare demographic data and characteristics (etiological diagnosis, type of medical specialty, day hospital visit, emergency and ward visit, and intensive care hospitalizations) of children and adolescents with PCDs followed in a University Hospital, according to the number of specialties/patient.

## METHOD

We performed a cross-sectional study with all PCD patients at the outpatient clinics of the Children’s Institute, Hospital das Clínicas of the School of Medicine at Universidade de São Paulo, followed during 2015. Patients treated exclusively by the emergency department, pediatric intensive care unit, and other hospitalizations were excluded. The Ethics Committee of our University Hospital approved this study.

PCDs were classified according to the duration of the disease (over three months), and the diagnosis was established by the physician’s scientific knowledge, valid methods or tools based on professional standards, and/or diagnostic classification criteria.^1^ ^,^ ^6^ The 10th Revision of the International Statistical Classification of Diseases and Related Health Problems (ICD-10) was also systematically evaluated to characterize the main PCD diagnoses.^6^ ^,^ ^21^ We assessed the following 23 pediatric specialties, according to the electronic data system: Allergy & Immunology, Cardiology, Endocrinology, Gastroenterology, Genetics, Hematology, Hematopoietic Cell Transplantation, Hepatology, Infectious Diseases, Nephrology/Renal Transplantation, Neurology, Nutrology, Oncology, Orthopedics, Palliative and Pain Care, Pediatric Surgery/Liver Transplantation, Pulmonology, Psychiatry, Rheumatology, and others (Adolescent Care, Preterm Care, and Pediatric Teaching Clinic).

We conducted data analyses using the electronic data system of institution, according to the number of physician appointments for PCDs. This study assessed: demographic data (current age, gender, and local of residence); characteristics of follow-up (duration, number of physician’s appointments/patient, and number of specialties/patient), types of pediatric specialty, number of day hospital clinic visits, and acute complications (number of emergency department visits, hospitalizations, intensive care unit admissions, and deaths). The etiology of each diagnosis was established based on ICD-10.

We divided the PCD patients into two groups, according to the number of pediatric specialties/patient: ≥3 specialties and ≤2 specialties.

The sample size provided a power of 80% to find differences of less than 2% in the two groups: ≥3 specialties and ≤2 specialties (GraphPad StatMate 1.01, GraphPad Software, Inc., CA, USA). The IBM-SPSS-22 software performed the statistical analyses. We presented the results as median (range) or mean±standard deviation (SD) for continuous variables and number (%) for categorical variables. Mann-Whitney test or Student’s t-test compared the continuous variables between the two study groups (≥3 specialties and ≤2 specialties). For categorical variables, the differences were evaluated by Fisher’s exact test. The multivariate analysis was carried out using backward stepwise logistic regression. In the regression model, the dependent variable was the presence of ≥3 specialties, and the independent variables were those with less than 20% significance level in the univariate analysis. For all statistical tests, p<0.05 was considered significant.

## RESULTS

From January to December 2015, 16,237 children and adolescents with PCDs were followed by 23 pediatric specialties in our Children’s Hospital.

Table 1 includes demographic data and characteristics of these children and adolescents with PCDs followed in a University Hospital, according to the number of specialties/patient. Patients followed by ≥3 specialties showed a significantly higher duration of follow-up compared to those followed by ≤2 specialties [2.1 (confidence interval of 95% - 95%CI 0.4-16.4) vs. 1.4 (95%CI 0.1-16.2) years; p<0.001], similar to the number of appointments in all specialties (≥3 specialties=24% vs. ≤2 specialties=3%; p<0.001). Frequencies of pediatric specialties, such as Infectious Disease (47 vs. 11%; p<0.001), Endocrinology (33 vs. 15%; p<0.001), Nephrology/Renal Transplantation (28 vs. 8%; p<0.001), and Pediatric Surgery/Liver Transplantation (28 vs. 14%; p<0.001), were significantly higher in the former group. We found no differences in patients followed by Oncology (Table 1). Patients followed by ≥3 specialties had significantly higher frequencies of day hospital clinic visits (7 vs. 2%; p<0.001), emergency department visits (45 vs. 21%; p<0.001), hospitalizations (35 vs. 15%; p<0.001), intensive care admissions (9 vs. 4%; p<0.001), and deaths (2 vs. 1%; p<0.001) (Table 2).

**Table 1:** Demographic data and characteristics of 16,237 children and adolescents with pediatric chronic diseases followed in a University Hospital, according to the number of specialties/patient.

	≥3	≤2	p-value
	(n=2,016)	(n=14,221)	
Demographic data			
Children (<10 years of age) [n (%)]	1,095 (54)	7670 (54)	0.76
Current age in years	9.0 (0.2-19.9)	9.1 (0-19.9)	0.67
Females [n (%)]	920 (46)	6,643 (47)	0.37
Residence in São Paulo [n (%)]	1,860 (92)	13,275 (93)	0.07
Duration of follow-up in years	2.1 (0.4-16.4)	1.4 (0.1-16.2)	<0.001
Physician appointment at outpatient clinics			
Number of appointments per patient			
1-3 [n (%)]	90 (4.5)	9,700 (68)	<0.001
4-12 [n (%)]	1,439 (71)	4,043 (28)	<0.001
≥13 [n (%)]	487 (24)	478 (3)	<0.001
Type of medical specialty			
Cardiology	303 (15)	460 (3)	0.008
Endocrinology	670 (33)	2,108 (15)	<0.001
Gastroenterology	444 (22)	641 (5)	<0.001
Genetics	475 (24)	976 (7)	<0.001
Hematology	205 (10)	541 (4)	<0.001
Hematopoietic cell transplantation	57 (3)	79 (1)	<0.001
Hepatology	519 (26)	1017 (7)	<0.001
Immunology and allergy	322 (16)	941 (7)	<0.001
Infectious disease	947 (47)	1,590 (11)	<0.001
Nephrology and renal transplantation	556 (28)	1,116 (8)	<0.001
Neurology	328 (16)	735 (5)	<0.001
Nutrology	192 (9)	215 (2)	<0.001
Oncology	251(13)	1,759 (12)	0.92
Orthopedics	8 (0.4)	10 (0.1)	0.001
Palliative and pain care	115 (6)	115 (1)	<0.001
Pediatric surgery and liver transplantation	564 (28)	2,018 (14)	<0.001
Pulmonology	504 (25)	994 (7)	<0.001
Psychiatry	98 (5)	90 (1)	<0.001
Rheumatology	191 (10)	590 (4)	<0.001
Others	570 (28)	1,708 (12)	<0.001

Results are presented in n (%), median (range), or mean±standard deviation.

**Table 2:** Characteristics of emergency department visits and hospitalization of 16,237 children and adolescents with pediatric chronic diseases followed in a University Hospital, according to the number of specialties/patient.

	≥3	≤2	p-value
	(n=2,016)	(n=14,221)	
Emergency department visits	908 (45)	2931 (21)	<0.001
≤2 [n (%)]	502 (25)	2145 (15)	<0.001
3-6 [n (%)]	281 (14)	655 (5)	<0.001
≥7 [n (%)]	125 (6)	131 (1)	<0.001
Hospitalizations [n (%)]	700 (35)	2,133 (15)	<0.001
1 [n (%)]	268 (13)	1182 (8)	<0.001
2 [n (%)]	1,244 (6)	446 (3)	<0.001
≥3 [n (%)]	308 (15)	505 (4)	<0.001
Intensive care unit admissions [n (%)]	173 (9)	511 (4)	<0.001
1 [n (%)]	120 (6)	372 (3)	<0.001
≥2 [n (%)]	53 (3)	139 (1)	<0.001
Day hospital clinic visits [n (%)]	142 (7)	222 (2)	<0.001
Deaths	39 (2)	101 (1)	<0.001
Age at death, years	7.3±5.8	7.6±5.6	0.78

Results are presented in n (%), median (range), or mean±standard deviation.

Table 3 illustrates the logistic regression analysis between the dependent variable (≥3 specialties) and the number of appointments, types of specialty, hospitalizations, intensive care unit admissions, and deaths. Psychiatry (*Odds Ratio* - OR=8.0; 95%CI 6-10.7; p<0.001), Palliative and Pain Care (OR=7.4; 95%CI 5.7-9.7; p<0.001), Infectious Disease (OR=7.0; 95%CI 6.4-7.8; p<0.001) and Nutrology (OR=6.9; 95%CI 5.6-8.4; p<0.001) were the medical areas with higher chances of being one of the three or more medical specialties. Logistic regression analysis demonstrated that PCD patients followed by ≥3 specialties were associated with higher risk of appointments/patient (>13) (OR=9.2; 95%CI 8.0-10.5; p<0.001), day hospital clinic visits (OR=4.8; 95%CI 3.8-5.9; p<0.001), emergency department visits (OR=3.2; 95%CI 2.9-3.5; p<0.001), hospitalizations (OR=3.0; 95%CI 2.7-3.3; p<0.001), intensive care admissions (OR=2.5; 95%CI 2.1-3.0; p<0.001), and deaths (OR=2.8; 95%CI 1.9-4.0; p<0.001) (Table 2).

**Table 3:** Multiple logistic regression analysis between the dependent variable (≥3 specialties) and the number of appointments, type of medical specialty, hospitalizations, intensive care unit admissions, and deaths.

	OR	95%CI	p-value
Number of appointments/patient			
1-3	0.02	0.18-0.3	<0.001
4-12	6.3	5.7-7.0	<0.001
≥13	9.2	8.0-10.5	<0.001
Type of medical specialty			
Cardiology	5.3	4.5-6.1	<0.001
Endocrinology	2.8	2.6-3.1	<0.001
Gastroenterology	5,9	5.2-6.8	<0.001
Genetics	4.2	3.7-4.7	<0.001
Hematology	2.8	2.4-3.4	<0.001
Hematopoietic cell Transplantation	5.2	3.7-7.3	<0.001
Hepatology	4.5	4.0-5.1	<0.001
Immunology and allergy	2.7	2.3-3.1	<0.001
Infectious disease	7.0	6.4-7.8	<0.001
Nephrology and renal Transplantation	4.5	4.0-5.0	<0.001
Neurology	3.6	3.1-4.1	<0.001
Nutrology	6.9	5.6-8.4	<0.001
Oncology	1.0	0.9-1.2	0.92
Orthopedics	-	-	-
Palliative and pain care	7.4	5.7-9.7	<0.001
Pediatric surgery and liver transplantation	2.3	2.1-2.7	<0.001
Pulmonology	4.4	3.9-5.0	<0.001
Psychiatry	8.0	6.0-10.7	<0.001
Rheumatology	2.4	2.0-2.9	<0.001
Others	-	-	-
Day hospital clinic visits	4.8	3.8-5.9	<0.001
Emergency department visits	3.2	2.9-3.5	<0.001
≤2	1.9	1.7-2.1	<0.001
3-6	3.4	2.9-3.9	<0.001
≥7	7.1	5.5-9.2	<0.001
Hospitalizations	3.0	2.7-3.3	<0.001
1	1.7	1.5-1.9	<0.001
2	2.0	1.6-2.5	<0.001
≥3	4.9	4.2-5.7	<0.001
Intensive care unit admissions	2.5	2.1-3.0	<0.001
1	2.4	1.9-2.9	<0.001
≥2	2.7	2.0-3.8	<0.001
Deaths	2.8	1.9-4.0	<0.001

OR: *Odds Ratio*; 95%CI: confidence interval of 95%.

Of note, we evaluated 106,437 appointments of 16,237 children and adolescents with PCD. The diagnosis of each PCD patient was only considered once, thus resulting in a total of 37.057 diagnoses. More than 2,500 ICD-10 were registered in PCD patients. The 20 most prevalent ICD-10 observed in PCD patients were: asthma (n=1538; 4.15%), short stature (n=1054; 2.84%), leukemia (n=501; 1.35%), obesity (n=435; 1.17%), malformation syndromes (n=429; 1.16%), transplant (n=420; 1.13%), urinary tract infection (n=384; 1.04%), epilepsy (n=306; 0.83%), hypothyroidism (n=286; 0.77%), pneumonia (n=282; 0.76%), chronic kidney disease (n=264; 0.71%), type 1 diabetes (n=248; 0.67%), chronic pain (n=243; 0.66%), diarrhea (n=244; 0.66%), malnutrition (n=214; 0.58%), constipation (n=214; 0.58%), juvenile idiopathic arthritis (n=186; 0.5%), high blood pressure (n=177; 0.48%), cystic fibrosis (n=174; 0.47%), and primary immunodeficiency (n=121; 0.33%).

Table 3 presents further comparisons of the 20 most prevalent associated diagnoses based on 37,057 ICD-10 descriptions, according to the number of specialties/patient. The diagnosis of asthma, obesity, chronic pain, transplant, urinary tract infection, pneumonia, chronic kidney disease, malnutrition, epilepsy, hypothyroidism, high blood pressure, diarrhea, constipation, and immunodeficiency were significantly higher in patients followed by ≥3 specialties compared to those followed by ≤2 specialties (Table 4).

**Table 4:** Comparison of the 20 most prevalent diagnosis based on 37,057 International Statistical Classification of Diseases and Related Health Problems descriptions, according to the number of specialties/patient.

	Number of specialties/patient		p-value
	≥3 (n=8,136) n (%)	≤2 (n=28,921) n (%)	
Asthma	383 (4.7)	1,155 (4.0)	0.005
Short stature	205 (2.5)	849 (2.9)	0.05
Obesity	134 (1.6)	301 (1.0)	<0.001
Chronic pain	123 (1.5)	120 (0.4)	<0.001
Transplant	116 (1.4)	304 (1.1)	0.006
Urinary tract infection	115 (1.4)	269 (0.9)	<0.001
Pneumonia	110 (1.4)	172 (0.6)	<0.001
Chronic kidney disease	106 (1.3)	158 (0.5)	<0.001
Malnutrition	105 (1.3)	109 (0.4)	<0.001
Epilepsy	92 (1.1)	214 (0.7)	0.001
Hypothyroidism	88 (1.1)	198 (0.7)	<0.001
High blood pressure	83 (1.0)	94 (0.3)	<0.001
Diarrhea	80 (1.0)	164 (0.6)	<0.001
Malformation syndromes	80 (1.0)	349 (1.2)	0.10
Constipation	73 (0.9)	141 (0.5)	<0.001
Primary immunodeficiency	48 (0.6)	73 (0.3)	<0.001
Leukemia	44(0.5)	457 (1.6)	<0.001
Type 1 diabetes	41 (0.5)	207 (0.7)	0.04
Cystic fibrosis	39 (0.5)	135 (0.5)	0.86
Juvenile idiopathic arthritis	36 (0.4)	150 (0.5)	0.42

Results are presented in n (%).

## DISCUSSION

The present study showed that PCD patients who required simultaneous care from multiple medical specialties had complex and severe diseases, with specific diagnoses.

The prevalence of PCDs has increased in the last half-century. Chronic diseases in pediatric populations that interfere with daily activities have increased more than 400% after 1960, resulting in greater medical complexity in clinical practice.^22^ Our study demonstrated that PCD patients followed by various medical specialties had high morbidity and mortality rates, with emergency department visits, hospitalizations, intensive care admissions, and deaths. These PCD patients needed frequent appointments at outpatient clinics, a fact that could lead to higher financial costs, requiring specific policies and interventions.^1^ ^,^ ^22^

Moreover, PCD patients of the present study followed by various medical specialties showed heterogeneity of diagnosis and subspecialties. Infectious Disease was the most frequent specialty for these patients, probably due to recurrent and severe infections, requiring multiple hospitalizations in immunosuppressed patients.^15^ ^,^ ^16^ ^,^ ^17^ ^,^ ^18^ Despite the relevant hospital-acquired infection committee, vaccination programs, and specific antibiotic and antifungal treatments for different PCDs in our University Hospital, patients might have many contributing factors for infections. These factors may be related to the disease itself (disease duration, lymphopenia, leukopenia, neutropenia, disease activity, functional asplenia, and primary immunodeficiencies) and its treatment (glucocorticoid and immunosuppressant agents).^13^ ^,^ ^15^ ^,^ ^16^ ^,^ ^18^ ^,^ ^23^ ^,^ ^24^

Endocrinology, Nephrology, Renal Transplantation, Pediatric Surgery, and Liver Transplantation were also frequent subspecialties in patients followed by more than three concomitant specialties. This fact results from a specific situation of our tertiary hospital, which is a reference center for the most common and severe endocrine, kidney, liver, and congenital diseases in Brazil.^14^ ^,^ ^16^ ^,^ ^25^

Importantly, Psychiatry and Palliative and Pain Care were the medical areas in which the patient had a great chance of being followed by three or more medical specialties. This finding is related to the medical complexities in our critically ill patients,^25^ ^,^ ^26^ who may also present several mental health conditions and chronic pain diseases.

Asthma was the main diagnosis associated with PCD patients followed by ≥3 specialties. The prevalence of this relevant diagnosis in the city of São Paulo ranges from 4.9 to 10.2%, similarly to our data.^27^ Additionally, 22% of adolescents reported wheezing symptoms.^28^ This chronic disease might lead to more frequent admission rates, requiring emergency department visits, hospitalizations, and intensive care admissions. Therapeutic optimization for PCD patients should be an important goal to attain.^13^ ^,^ ^27^

Short stature was also relevant herein, a multifactorial finding related to long disease duration, chronic inflammatory diseases, physical inactivity, and therapies (such as glucocorticoid). In addition, Endocrinology and Nutrology were significant specialties due to the increase in obesity in the general population, as well as in chronic disease patients.^29^

Pediatric care systems and multidisciplinary health teams should develop evidence-based solutions to the challenges of caring for and treating PCD patients with a medically complex disease.^23^ Strategies should be devised focusing on reducing hospital admission rates, thus improving hospital care for these patients.

The strong point of this study was the large population with different PCDs defined by ICD-10 descriptions and followed in a tertiary and pediatric teaching hospital. Our university hospital is a Brazilian reference center for pediatric and complex specialties that follows PCDs. This center is well known for its high standard and humane care, has many health care providers, modern resources with clinical/laboratory research and drug trials, evaluating children and adolescents with medically complex conditions.^1^ ^,^ ^15^ ^,^ ^16^ The main limitations of the present study were the short evaluating period and its cross-sectional design.

In conclusion, the present study showed that PCD patients who needed care from multiple medical specialties had complex and severe diseases. PCD patients presented specific diagnoses, particularly asthma, short stature, and leukemia
